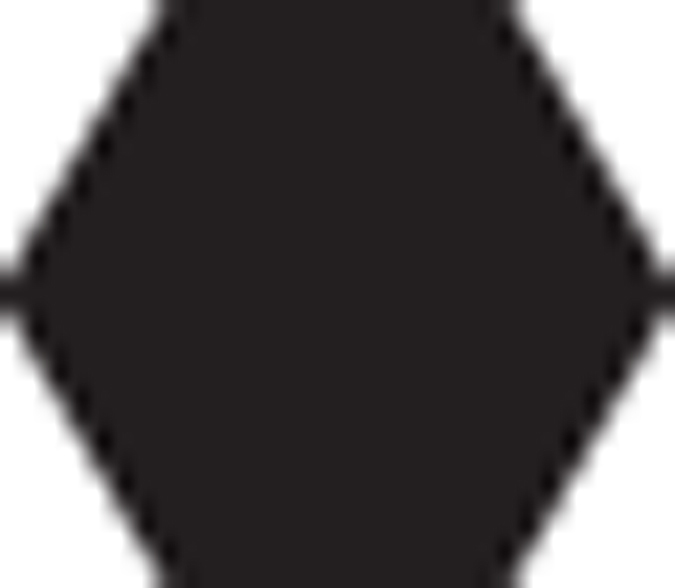# TSPO ligands stimulate ZnPPIX transport and ROS accumulation leading to the inhibition of *P. falciparum* growth in human blood

**DOI:** 10.1038/srep33516

**Published:** 2016-09-19

**Authors:** I. Marginedas-Freixa, C. Hattab, G. Bouyer, F. Halle, A. Chene, S. D. Lefevre, M. Cambot, A. Cueff, M. Schmitt, B. Gamain, J. J. Lacapere, S. Egee, F. Bihel, C. Le Van Kim, M. A. Ostuni

**Affiliations:** 1 UMR_S1134, Université Sorbonne Paris Cité, Université Paris Diderot, Inserm, INTS, Unité Biologie Intégrée du Globule Rouge, Laboratoire d’Excellence GR-Ex, Paris, France; 2 UMR 8227 Comparative Erythrocyte’s Physiology, CNRS, Université Pierre et Marie Curie, Sorbonne Universités, Laboratoire d’Excellence GR-Ex, F-29680 Roscoff, France; 3 UMR7200, Faculty of Pharmacy, University of Strasbourg, CNRS, 67400 Illkirch Graffenstaden, France; 4 UMR 7203 LBM, CNRS, Université Pierre et Marie Curie, Sorbonne Universités, École Normale Supérieure - PSL Research University, Département de Chimie. F-75005 Paris, France

## Abstract

After invading red blood cells (RBCs), *Plasmodium falciparum* (*Pf*) can export its own proteins to the host membrane and activate endogenous channels that are present in the membrane of RBCs. This transport pathway involves the Voltage Dependent Anion Channel (VDAC). Moreover, ligands of the VDAC partner TranSlocator PrOtein (TSPO) were demonstrated to inhibit the growth of the parasite. We studied the expression of TSPO and VDAC isoforms in late erythroid precursors, examined the presence of these proteins in membranes of non-infected and infected human RBCs, and evaluated the efficiency of TSPO ligands in inhibiting plasmodium growth, transporting the haem analogue Zn-protoporphyrin-IX (ZnPPIX) and enhancing the accumulation of reactive oxygen species (ROS). TSPO and VDAC isoforms are differentially expressed on erythroid cells in late differentiation states. TSPO2 and VDAC are present in the membranes of mature RBCs in a unique protein complex that changes the affinity of TSPO ligands after *Pf* infection. TSPO ligands dose-dependently inhibited parasite growth, and this inhibition was correlated to ZnPPIX uptake and ROS accumulation in the infected RBCs. Our results demonstrate that TSPO ligands can induce *Pf* death by increasing the uptake of porphyrins through a TSPO2–VDAC complex, which leads to an accumulation of ROS.

Malaria is an endemic infectious disease that is caused by various species of the protozoa genus *Plasmodium.* It remains a major public health problem in developing countries worldwide, resulting in approximately 200 million new infections per year and more than 500,000 subsequent deaths^1^.

*Plasmodium falciparum* (*P. falciparum*) infection induces dramatic modifications in the plasma membrane of the host red blood cell (RBC) by exporting its own proteins that modify RBC properties and rheology^2^.

Since the discovery of quinine at the beginning of the 19^th^ century, different drugs have been synthesised to improve malaria treatment^3^. However, the regular appearance of drug-resistant parasite strains is difficult to overcome when treating patients with malaria^3^.

In this sense, the discovery and chemical development of new molecules able to compromise the viability or transmission of the parasite (alone or combined with well-known antimalarial drugs) are necessary to develop novel treatments. Moreover, understanding the mechanisms of action and identifying the biological targets of novel molecules are crucial for improving the efficacy of parasite growth inhibition.

Translocator Protein (TSPO) is an 18 kDa ubiquitous protein that was previously known as peripheral benzodiazepines receptor (PBR). It has been shown to integrate a mitochondrial membrane complex of approximately 800 kDa with other protein partners such as the voltage-dependent anion channel (VDAC) or the adenine nucleotide transporter (ANT), which exhibits a cholesterol transport function involved in steroidogenesis^4^,^5^. TSPO has also been shown to be involved in the transport of other molecules such as porphyrins^6^,^7^,^8^,^9^. These transport functions can be modulated by TSPO ligands, including benzodiazepines such as Ro5-4864, diazepam and flunitrazepam, isoquinolines such as PK 11195, and pyridazinoindoles such as SSR-180,575^10^,^11^.

To date, two human TSPO isoforms have been identified. The most investigated, TSPO1, is ubiquitously present on the outer mitochondrial membrane of cells in most tissues. Over the last two years, different TSPO1 structures were determined, demonstrating five transmembrane helices that are highly conserved among species^12^,^13^,^14^,^15^. The second isoform, TSPO2, was recently identified and proposed to be specifically localised to the nuclear and plasma membranes and at the endoplasmic reticulum of erythroid cells^16^, but the specific function of the isoform is not clear.

Previous studies have demonstrated that TSPO^17^,^18^,^19^,^20^ and VDAC^17^,^18^,^20^,^21^ are present in the membrane of RBCs, and we and others have proposed that *P. falciparum* infection induces an increase in RBC membrane conductance through VDAC-like channel activity^17^,^22^. Despite the observations that adding micromolar concentrations of TSPO ligands to the culture medium impairs *Plasmodium* growth, thereby inducing parasite death^17^,^22^, the mechanisms leading to this action are still unknown.

In the present study, we examined the expression of TSPO isoforms and VDAC during the late erythroid differentiation stages. We also characterised the membrane complexes containing these proteins in healthy and infected mature RBCs and investigated the mechanisms of action leading to the inhibition of parasite growth by TSPO ligands. We examined the effects of three TSPO ligands belonging to different chemical families. Interestingly, the isoquinoline PK 11195 binds in a pocket located between the five transmembrane helices^12^,^14^, whereas the benzodiazepine Ro5-4864 binds to TSPO in its complexed form^23^.

The compounds tested herein, which exhibited nanomolar affinities for TSPO1, possessed micromolar affinities for RBC membranes and significantly inhibited parasite growth. Moreover, they stimulated zinc protoporphyrin-IX (ZnPPIX) transport in a dose-dependent manner and increased reactive oxygen species (ROS) accumulation.

Our results led us to propose a model by which TSPO ligands induce the uptake of the haem structural analogue ZnPPIX, leading to the death of the parasite due to increased intracellular oxidative stress. Interestingly, this mechanism does not target the parasite *per se* but most likely improves the capability of RBCs to protect themselves against the parasite, thus limiting the development of drug resistance.

## Results

### TSPO1 and 2 isoforms are differentially expressed during *in vitro* erythroid differentiation

**Figure 1:**
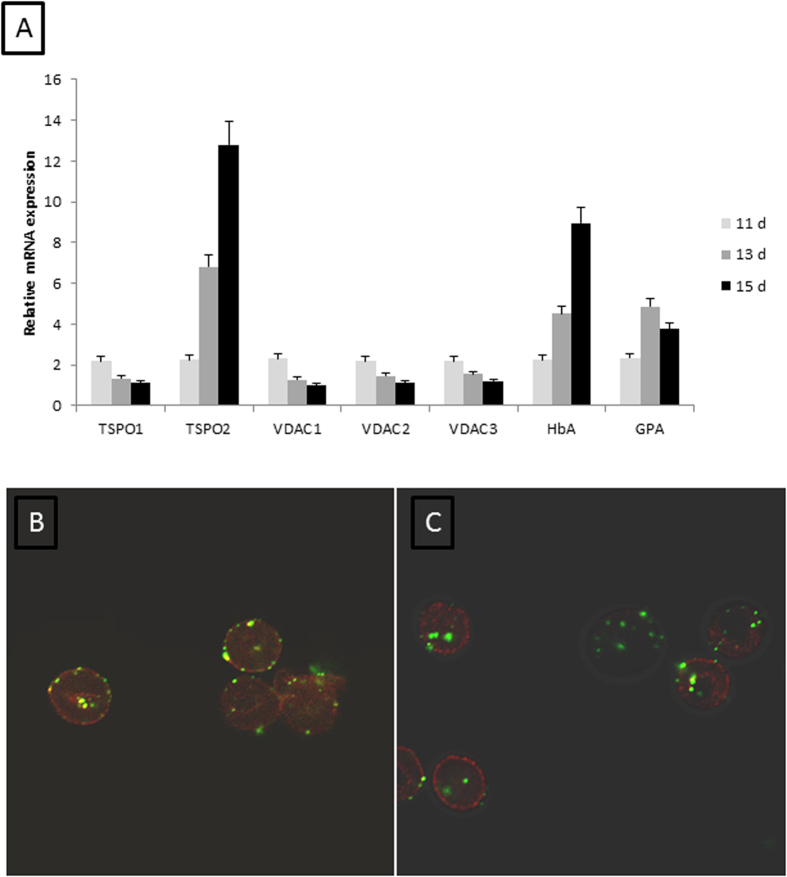
q-RT-PCR of mRNA isolated from culture human CD34+ cells at different differentiation states and Immunofluorescence staining of mature RBCs. The expression of TSPO1, TSPO2, VDAC1, VDAC2, VDAC3, α-chain haemoglobin (HbA) and glycophorin A (GpA) was measured by q-RT-PCR at days 11, 13 and 15 of differentiation (**A**). The changes in specific mRNA levels were calculated using the ΔΔ*CT* method (where *CT* is the threshold cycle), with results presented as the mean ± SEM. The results were normalised to PABPC1 gene expression. Triplicate analyses were performed for each target gene. Immunofluorescence staining against TSPO2. VDAC1, -2, -3 was performed on mature RBCs. Confocal stacking images show that TSPO2 (panel B, in green) and VDAC1, -2, -3 (panel C, in green) are present in the membrane of human RBCs. Anti-GpA antibody was used to label RBC membrane (panels B and C, in red).

CD34^+^ cells were subjected to *in vitro* erythroid differentiation, following the procedure described by Giarratana *et al*.^24^,^25^, leading to a major erythroid commitment from day 4 of differentiation and an exclusive production of mature erythroid cells at day 18 of the culture*. In vitro* erythroid differentiation was controlled by cytological analysis (Supplementary Figure 1), erythroid stage counts (Supplementary Table 1) and expression of specific markers glycophorin A (GPA) and haemoglobin A (HbA) (Fig. 1A).

The expression of TSPO and VDAC isoforms was analysed in cultures by RT-qPCR at days 11, 13 and 15 of *in vitro* differentiation. TSPO1 expression decreased throughout the differentiation phase, whereas TSPO2 expression increased greatly (Fig. 1A). At the same time, the expression of the well-known TSPO partner VDAC decreased in the same manner as TSPO1 (Fig. 1A).

### TSPO2 and VDAC are involved in human RBC membrane complexes

Specific antibodies raised against different epitopes of TSPO1 and TSPO2 (Supplementary Figure 2) were used to assess the presence and polymeric state of TSPO and VDAC in RBC membranes.

TSPO2 and VDAC are expressed on the plasma membrane of mature erythrocytes, as revealed by immunofluorescence experiments (Fig. 1B,C, Supplementary Figure 3).

**Figure 2:**
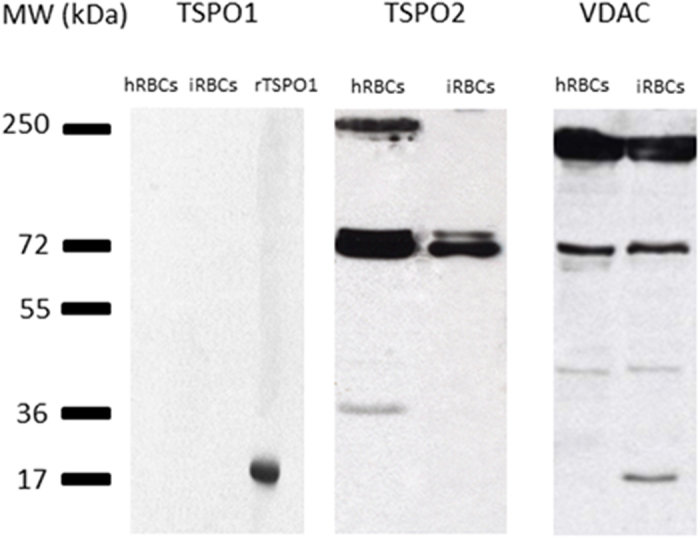
Polymeric forms of TSPO2 and VDAC on red blood cell membranes. Samples of healthy (hRBC) and infected (iRBC) red blood cell membranes (ghosts) were analysed under denaturing conditions after SDS lysis. TSPO2 was primarily found in a tetrameric form at 72 kDa with a minor dimeric form at 36 kDa that was only present in hRBCs. VDAC was primarily identified in a dimeric form of 68 kDa, with another dimeric form of 58 kDa only present in hRBCs and a truncated form of 17 kDa only detected in iRBCs. There was no immunoreaction against TSPO1 in RBCs. Recombinant human TSPO1 (rTSPO1) was used as an antibody positive control, yielding a band at the expected size of 21 kDa.

Under conventional SDS-PAGE conditions, we did not observe the presence of the TSPO1 isoform, but we did detect both TSPO2 and VDAC (Fig. 2). The molecular weights observed for TSPO2 suggested the presence of covalent tetrameric and dimeric forms (72 and 36 kDa). This was previously reported for TSPO1, which was able to form covalent functional oligomers, even under denaturising conditions^26^. In this report, authors described the expression of TSPO in two different cell lines, MA10-Leydig and MA231-breast cancer, and showed that TSPO is expressed only in a dimeric form in MA231 and mainly in the dimeric form compared to the trimer and the monomer in MA10-Leydig cells. Furthermore, there is abundant literature showing that TSPO isoforms are usually present in oligomeric, SDS-resistant forms^16^,^26^,^27^,^28^. The TSPO partner VDAC was found principally in a dimeric form of 68 kDa, as previously described^17^.

Analysis of RBC membranes from *P. falciparum*-infected RBCs demonstrated that the TSPO2 and VDAC polymeric states were not affected by the entry of the parasite into the RBC (Fig. 2).

Only our previous paper^17^ and two recent publications^18^,^20^ have described the presence of TSPO and VDAC isoforms in RBC membranes using mass spectrometry proteomics. However, both the number of peptides detected and the coverage rate are very low compared to those of other well-characterised and abundant proteins. We isolated the TSPO2 and VDAC major immunoreactive bands with a molecular weight between 56 and 72 kDa, which corresponded to TSPO2 tetramers and/or a VDAC dimer, and we analysed them by mass spectrometry. The results summarised in Table 1 show that TSPO2 and three isoforms of VDAC were present in those bands, as were four isoforms of ANT. Additional information about the matching peptides is summarised in Supplementary Dataset 1.

It was previously proposed that differences in the mass spectrometry results among different publications could originate from traces of platelet or white blood cell (WBC) contaminants^18^. FACS analysis using platelet (anti-CD61) and WBC (anti-CD45) specific markers showed that, after leucodepletion and washing, the concentrated RBC samples contained less than 8 WBC and 20 platelets per million RBCs (Supplementary Table 2).

**Figure 3:**
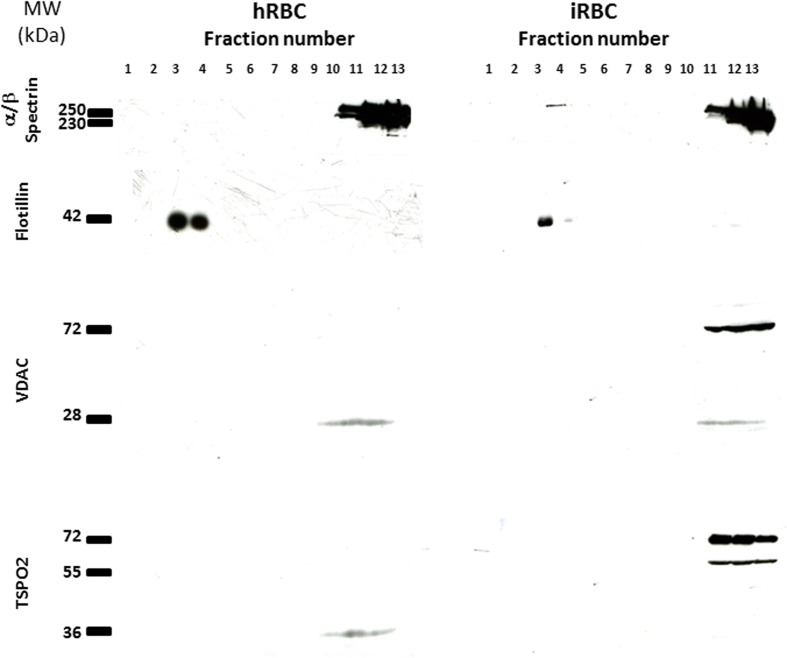
Localisation of TSPO2 and VDAC in RBC membrane microdomains. Samples of healthy (hRBC) and infected (iRBC) red blood cells were lysed with 1% Triton X-100 and separated by ultracentrifugation in a sucrose gradient. Immunoreactivity against α/β spectrin, flotillin-2, VDAC and TSPO2 were analysed under denaturing conditions. Both TSPO2 and VDAC were located in the α/β-spectrin positive fractions.

The presence of high molecular weight bands (230–250 kDa), which were also recognised by an anti-spectrin antibody (Supplementary Figure 4), suggested a possible association of TSPO2 and VDAC with this skeletal protein. To study such an interaction, we used a well-characterised protocol to determine the distribution of TSPO2 and VDAC in RBC membrane microdomains. hRBCs or iRBCs were solubilised using Triton X-100 and separated by ultracentrifugation in a sucrose gradient. The immunoblotting results showed that TSPO2 and VDAC were located in spectrin-positive heavy fractions in both hRBCs and iRBCs (Fig. 3). These results could indicate an interaction with the RBC membrane skeleton. Neither TSPO2 nor VDAC were found in the fractions positive to flotillin-2, a protein marker of RBC lipid rafts^29^. Interestingly, only the monomeric and dimeric forms of both partner proteins were present in hRBC fractions, whereas higher-order polymeric forms were primarily detected in iRBCs, even when the membrane fractions were treated with Triton X-100.

Our results showed that TSPO2 and VDAC were both present in the same RBC microdomains, which is in agreement with previously published data indicating that TSPO1 is involved in high molecular weight mitochondrial complexes, together with VDAC and other membrane partners^5^,^30^. To confirm the presence of multimeric complexes involving VDAC and TSPO2 in the hRBC membranes analysed in native conditions, we used well-described mild detergent solubilisation protocols^31^. Both TSPO2 and VDAC were found in a complex of 800 kDa (Supplementary Figure 5).

### Plasmodium infection induces changes in the affinity of TSPO ligands

**Figure 4:**
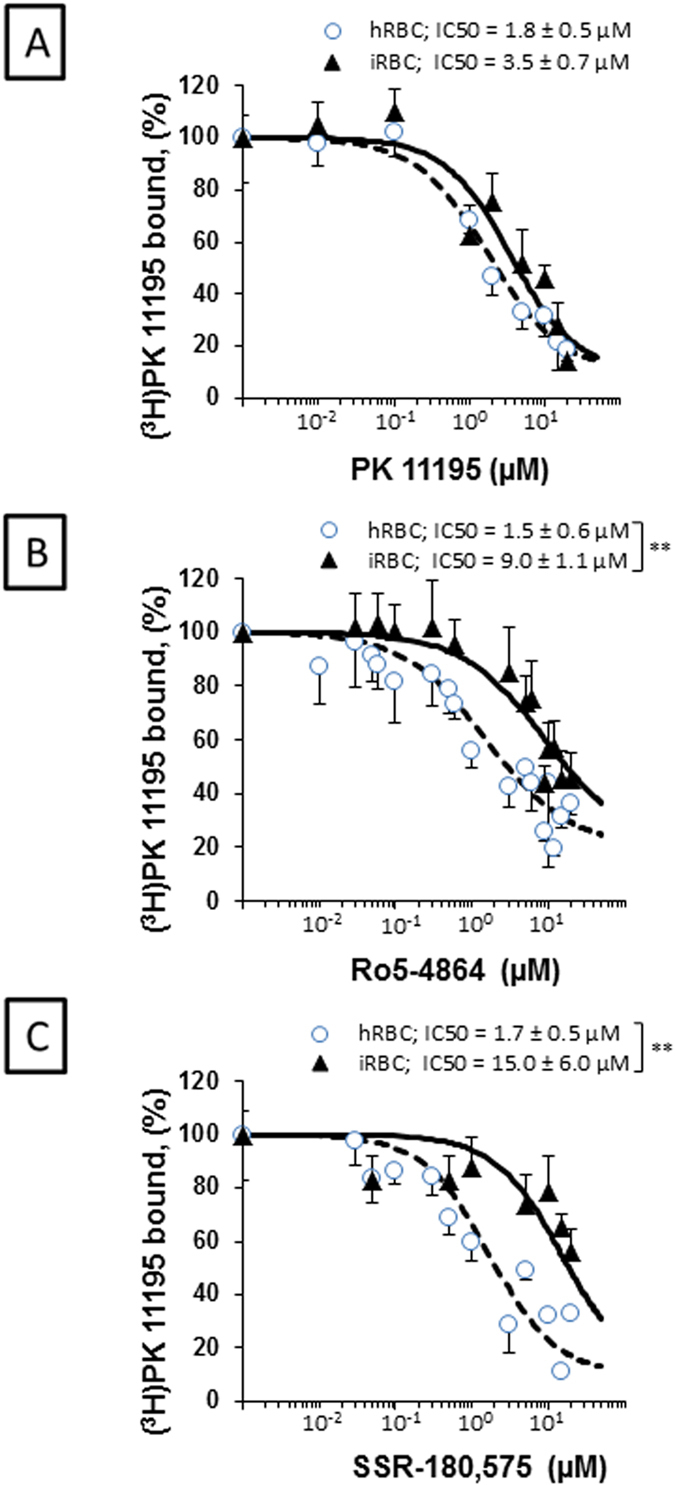
TSPO ligands affinity for healthy (hRBC) and infected (iRBC) red blood cell membranes. hRBCs and iRBCs were incubated in the presence of (^3^H)PK 11195 and increasing concentrations of non-radiolabelled **(A)** PK 11195; **(B)** Ro5-4864 and **(C)** SSR-180,575. iRBCs were present in both ring and thropozoite stages; no differences in parasite stages were seen. IC_50_ values indicated in the respective panels were calculated from displacement curves using the following equation: *Y* = 100 × (*S*)^*h*^/(IC_50_h* *× *S*^*h*^), where Y is the percentage of bound [^3^H] PK 11195, *S* is the unlabelled ligand concentration, and *h* is the Hill coefficient (1 ± 0.2). Data are presented as the mean ± SEM. Each curve represents the mean of at least 3 experiments. IC_50_ values were considered significantly different at *p < 0.05; **p < 0.01.

Because the TSPO ligand affinity could be dependent on the constitution of the TSPO complex^23^,^32^, we assessed whether the infection was followed by changes in ligand affinity. All ligands were found to possess an affinity for the membranes in both non infected or infected conditions on a micromolar level, but infection induced a significant loss of affinity (p < 0.01) for the Ro5-4864 and SSR-180,575 compounds (Fig. 4). The loss of affinity was not dependent on the stage of the parasite.

### TSPO ligands induce parasite growth’s inhibition

Previous studies have demonstrated that the TSPO ligands PK 11195, Ro5-4864, diazepam and flurazepam are able to inhibit *P. falciparum* growth, most likely by diminishing parasite-induced conductance, thus leading to parasite death^17^,^22^. In this study, we tested 3 well-known TSPO specific ligands to determine the pathways leading to parasite death.

**Figure 5:**
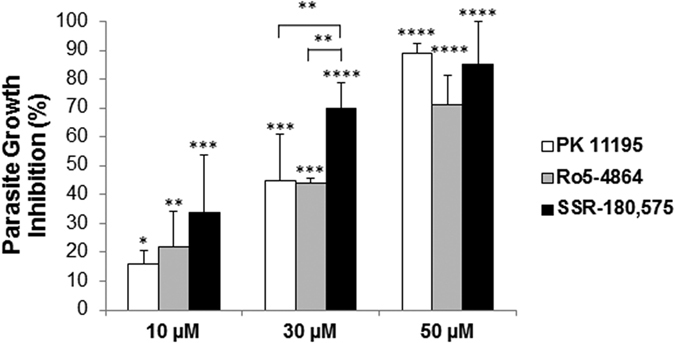
Parasite growth inhibition induced by TSPO ligands. Infected red blood cells (iRBCs) synchronised in ring stages were diluted to 1% parasitemia and cultured at 5% haematocrit in the presence of TSPO ligands. Parasitemia was maintained between 1 and 5% by dilution at 48 h, together with medium and TSPO ligand renewal. Percentages of growth inhibition were obtained after 2 parasite life cycles via flow cytometry analysis and were normalised to the control condition (solvent treated cells). Data are presented as the mean ± SEM; n = 6. Statistical analyses were performed for each ligand concentration and each control condition as well as between ligands. Differences were considered significantly different at *p < 0.05; **p < 0.01; ***p < 0.001; ****p < 0.0001.

The growth of *P. falciparum* was assessed by flow cytometry analysis of parasitemia after culture in the presence of TSPO ligands for two parasite cycles (96 h). Parasitemia was maintained between 1 and 5% to allow exponential parasite growth throughout the assay. All 3 ligands dose-dependently inhibited parasite growth (Fig. 5), reaching 80–100% growth inhibition at a concentration of 50 μM. Moreover, the pyridazinoindole SSR-180,575 exhibited the strongest anti-plasmodium effect at a concentration of 30 μM, exhibiting 70% parasite growth inhibition (p < 0.01 over PK 11195 and Ro5-4864). Interestingly, all ligands were capable of inducing parasite death *in vitro* without any detectable effect on the haemolysis of RBCs (Supplementary Table 3).

### Zinc-Protoporphyrin IX (ZnPPIX) uptake is modulated by TSPO ligands

Mitochondrial TSPO has been previously shown to be involved in the transport of porphyrins and haem analogues^7^,^8^,^9^. In the current study, we sought to assess the possibility that a similar function occurs at the RBC membrane. We measured the uptake of ZnPPIX, a fluorescent haem analogue that has previously been shown to enter iRBCs^33^.

**Figure 6:**
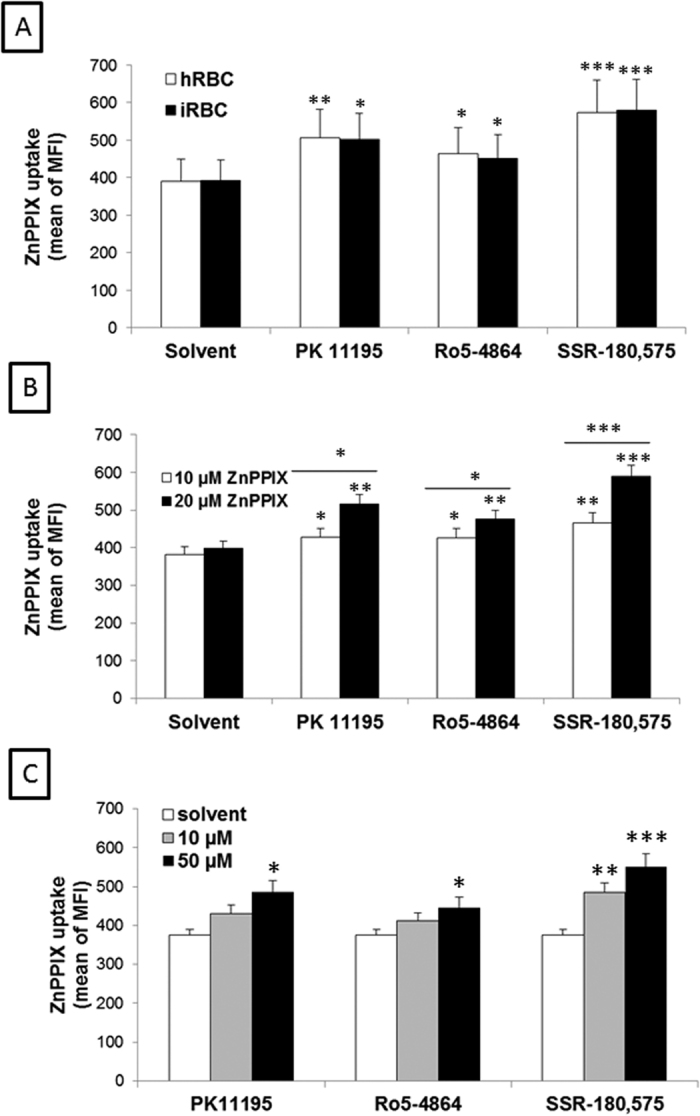
ZnPPIX uptake in healthy (hRBC) and infected (iRBC) red blood cells. iRBCs at 2 to 5% parasitemia and hRBCs were incubated under different conditions and the uptake of ZnPPIX was assessed at the time point of 8 h by flow cytometry. MFI values were normalised to background levels for control and ligand treated conditions. **(A)** Incubation in the presence of 20 μM ZnPPIX and 50 μM TSPO ligands. **(B)** Incubation in the presence of 50 μM TSPO ligands and 10 or 20 μM ZnPPIX. **(C)** Incubation in the presence of 20 μM ZnPPIX and 10 or 50 μM TSPO ligands. Data are presented as the mean ± SEM; n = 7. Statistical analyses were performed for each ligand concentration and each control condition as well as between ligands. Differences were considered significantly different at *p < 0.05; ** p < 0.01; ***p < 0.001; ****p < 0.0001.

ZnPPIX was incorporated into both hRBCs and iRBCs under control conditions, and this process was significantly increased by the addition of TSPO ligands to the culture media (Fig. 6A). This uptake was dependent on initial concentration of ZnPPIX (Fig. 6B) as well as on the ligand’s concentration (Fig. 6C). The kinetics of ZnPPIX uptake were slow, and the difference between RBCs treated with TSPO and untreated RBCs became significant only after 8 h of incubation, reaching saturation after 18 h (Supplementary Figure 6). Among the three ligands tested, SSR-180,575 appeared to be the most efficient at increasing the entry of ZnPPIX, demonstrating a 1.48-fold increase at 8 h (p < 0.01) in ZnPPIX uptake versus a 1.28-fold (p < 0.05) and 1.15-fold (p < 0.05) increase in uptake associated with PK 11195 and Ro5-4864, respectively.

None of the tested TSPO ligands demonstrated cytotoxic effects when added at concentrations up to 50 μM in non-infected or infected RBCs (Supplementary Table 3) in either the presence or absence of ZnPPIX. However, the addition of the haem analogue systematically induced a slight increase in the rate of haemolysis.

### ZnPPIX uptake induced by TSPO ligands is not related with changes in erythrocyte membrane fragility

Using the sorbitol-induced haemolysis assays, we previously demonstrated that TSPO ligands are able to directly modulate RBC membrane transport^17^. The present results confirm this specific and concentration-dependent action of TSPO ligands (Supplementary Figure 7) and show that Ro5-4864 is more effective than PK 11195 or SSR-180,575 (p < 0.01).

**Figure 7:**
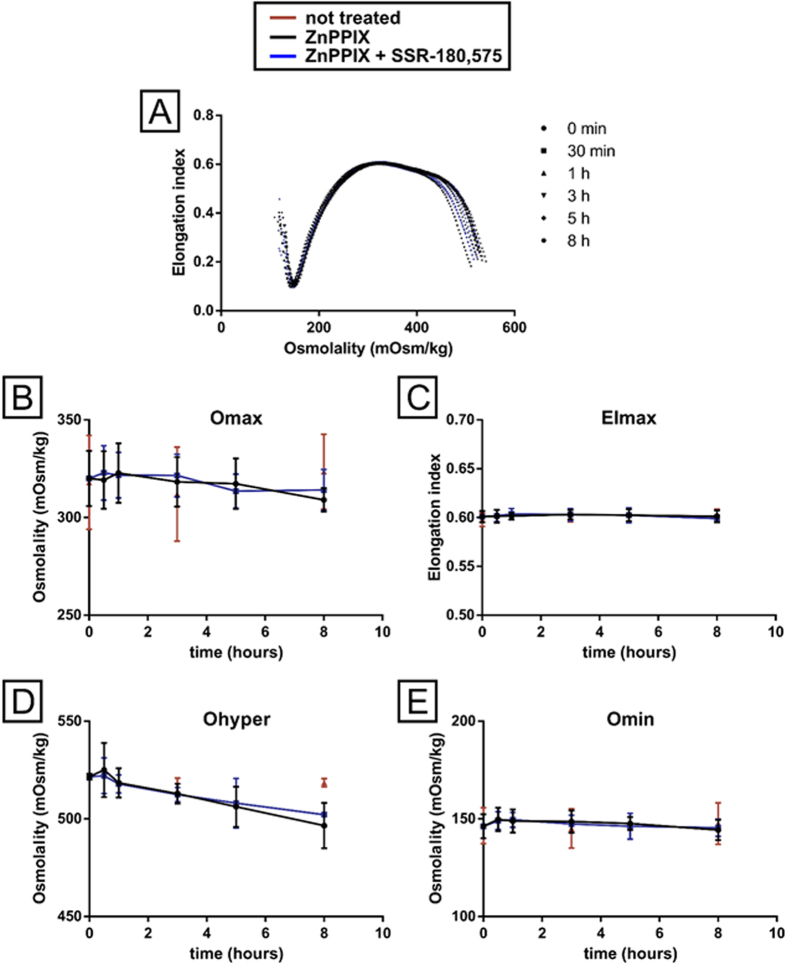
Effect of ZnPPIX and SSR-180,575 on RBC membrane fragility. RBCs were incubated with 20 μM ZnPPIX with or without 50 μM SSR-180,575 for up to 8 h. At different incubation times, RBC samples were studied by ektacytometry under osmotic stress to assess RBC deformability under an osmotic gradient. **(A)** Representative ektacytometer curves at different incubation times (● 0 min; ■ 30 min; ▲ 1 h; ▼ 3 h; ♦5 h and ⬣ 8 h) in control conditions (red symbols), in the presence of ZnPPIX (black traces) or ZnPPIX + SSR-180,575 (blue traces). **(B–E)** Control conditions (red traces) and the impact of ZnPPIX (black traces) or ZnPPIX + SSR-180,575 (blue traces) on: **(B)** the osmolality associated with the maximal elongation index (O_max_); **(C)** the maximal elongation index (EI_max_); **(D)** the high osmolality associated with the half of the EImax (O_hyper_) and **(D)** the osmolality associated with the lowest deformability (O_min_). Data are presented as the mean ± SEM; n = 4.

Nevertheless, one can suggest that in the presence of ZnPPIX, this ligand-modulated transport could be partially or entirely related to altered membrane fragility. We performed a well-characterised osmoscan analysis to assess the effect of the TSPO ligand SSR-180,575, with or without ZnPPIX, on osmotic fragility in healthy RBC at several incubation times (30 min to 8 h). The results are summarised in Fig. 7 and show that neither ZnPPIX nor SSR-180,575, alone or together, affected RBC deformability in response to osmotic challenge. We analysed the four parameters that are usually measured to assess osmotic fragility: osmolality allowing the maximal deformability (O_max_, Fig. 7B); maximal elongation index (EI_max_, Fig. 7C); low osmolality giving the minimal deformability (Omin, Fig. 7D); and high osmolality giving the half of maximal deformability (O_hyper_, Fig. 7E). Only the O_hyper_ parameter was affected compared to non-treated cells after an 8-h incubation, which is consistent with ZnPPIX import.

### ZnPPIX uptake is coupled to enhanced ROS accumulation

**Figure 8:**
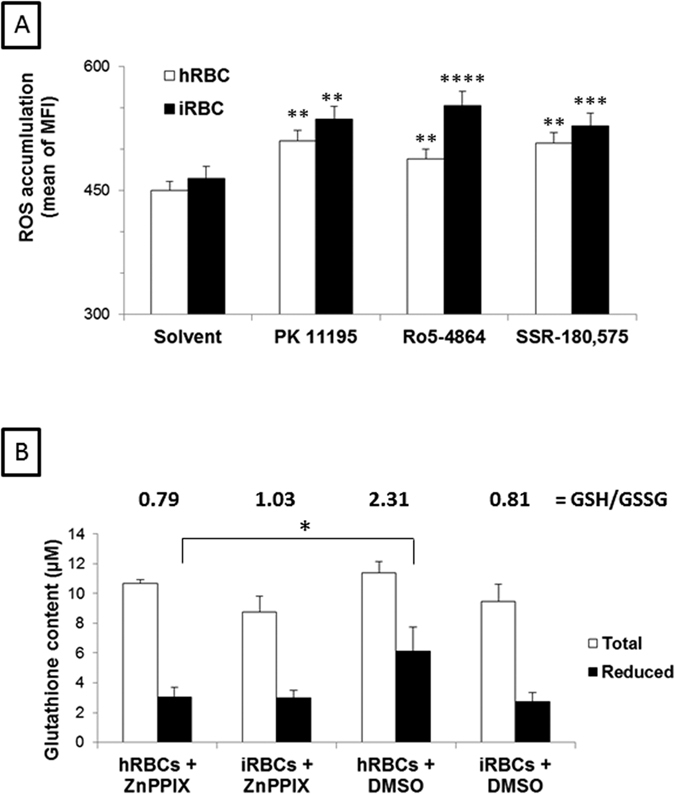
Reactive oxygen species (ROS) accumulation and glutathione content in infected (iRBC) and healthy (hRBC) red blood cells after ZnPPIX uptake. (**A**) iRBC at 2 to 5% parasitemia and hRBCs were incubated in media containing 20 μM ZnPPIX and 50 μM TSPO ligands. After 4.5 h, samples were washed and incubated with DCFDA and analysed via flow cytometry. No compensation was needed as monostained samples showed no interference between the ZnPPIX and ROS probes. (**B**) Alternatively, iRBCs and hRBCs were incubated in a media containing 20 μM ZnPPIX or DMSO for 2.5 h, followed by the analysis of glutathione content. Values correspond to the ratio of GSH/GSSG. Data are presented as the mean ± SEM; n = 8. Differences between each ligand and each control condition were considered significantly different at *p < 0.05; **p < 0.01; ***p < 0.001; ****p < 0.0001.

The addition of ZnPPIX to the culture media of iRBCs and hRBCs induced an increase in the accumulation of ROS that was significantly increased by the addition of the three TSPO ligands (p < 0.01 for PK 11195 and SSR-180,575; p < 0.005 for Ro5-4864) (Fig. 8A). ROS accumulation peaked 4.5 h after ZnPPIX addition and slowly decreased after the peak (Supplementary Figure 8A).

ROS accumulation was also observed after incubation of iRBCs in the presence of TSPO ligands, in spite of a lack of ZnPPIX in the medium (Supplementary Figure 8B). However, under these conditions, the process was slower than in the presence of the haem analogue. The ROS peak was reached only 12 h after the addition of the ligands.

These results demonstrated that TSPO ligands induced a constitutive ROS accumulation in both hRBCs and iRBCs and that this phenomenon was accelerated by the presence of ZnPPIX.

We investigated a putative difference between hRBC and iRBC in regards to basal antioxidant status that could reflect the sensitivity of the parasite to oxidative stress. We measured the amounts of glutathione, which is the main antioxidant in RBCs, and how its levels were regulated in the presence of ZnPPIX in the culture media.

As shown in Fig. 8B, the entry of the parasite in the RBCs was associated with a decrease in the total amount of cellular glutathione and a decrease in the reduced/oxidised (GSH/GSSG) ratio (P < 0.05), indicating that a pro-oxidant effect was induced by parasite entry. The accumulation of ZnPPIX in hRBCs also exerted a pro-oxidant effect, as observed by a decrease in both the level of GSH and the GSH/GSSG ratio. In contrast, ZnPPIX accumulation in iRBCs was not able to induce an additional decrease in the levels of total, reduced, or oxidised glutathione, most likely because the glutathione system had already been diminished by parasite entry^34^.

## Discussion

More than two decades ago, two different research groups described the presence of TSPO binding sites on the RBC membrane using radiolabelled TSPO specific ligands^19^,^35^. At that time, only TSPO1 was identified as a mitochondrial outer membrane protein involved in different functions that could be modulated by specific TSPO ligands. These functions included haem transport and apoptosis^9^,^11^,^36^,^37^. The recent discovery of a new TSPO isoform, named TSPO2^16^,^38^ and described as specific to the erythroid lineage, raised questions concerning the identity of the TSPO isoform/s present in the RBC membrane, the assembly of membrane complexes involving TSPOs, the role of this protein in the RBC and the ability of this protein to interact with the ligands of reference. Some of these questions were partially answered in our previous study^17^, in which we demonstrated the expression of two well-characterised TSPO partners in the RBC membrane: the voltage dependent anion channel (VDAC) and the adenine nucleotide transporter (ANT). We also reported that primary cultured erythroid cells expressed mRNA of both TSPO isoforms and that RBC membranes were immunoreactive against TSPO. However, the antibody used for those studies did not enable us to distinguish between the two TSPO isoforms.

The present results demonstrate that both TSPO isoforms are expressed during *in vitro* erythroid maturation; the expression of TSPO2 is mainly activated during the EPO-induced differentiation phase and is accompanied by a repression of TSPO1 expression. After the differentiation process, TSPO2 is the main isoform retained in mature RBC membranes, where it is mainly found in tetramers. TSPO2 is associated with VDAC in a multimeric membrane complex of 800 kDa. Interestingly, in mitochondria, TSPO1 also forms a complex with VDAC^5^, suggesting the presence of conserved interacting binding sites. Furthermore, our results suggested that TSPO2 and VDAC are located in RBC membrane microdomains together with spectrin, supporting the hypothesis that TSPO2/VDAC in membrane complexes interacts with the erythroid membrane skeleton.

In agreement with previously published results^17^,^22^, we also found that micromolar concentrations of TSPO ligands are necessary to significantly inhibit *P. falciparum* growth. Such values contradict the well-characterised TSPO ligand affinities that are usually found in the nanomolar range for the TSPO1 isoform^19^,^35^,^39^. This discrepancy could be explained by the fact that TSPO2 is the main isoform located in the RBC membrane; as previous reports have indicated that this isoform presents high nanomolar affinity for cholesterol but a dramatically diminished affinity for PK 11195^16^. We demonstrate that *P. falciparum* infection induces a significant diminution of TSPO ligand affinity. This affinity change could be the result of changes in the membrane protein complex composition that are induced by the parasite. An alternative but not exclusive hypothesis could be that parasite infection favours the covalent union of complex proteins. This is in agreement with previous reports indicating that TSPO ligand affinities depend on TSPO complex composition^23^,^32^ and that TSPO polymerisation decreases PK 11195 affinity^26^.

After uncovering the need for high ligand doses, we aimed to study the mechanisms of action involving TSPO ligand binding that lead to *P. falciparum* growth inhibition. Because the TSPO2 functions were not fully understood, we first tested whether TSPO ligands were able to inhibit haem transport through the RBC membrane, in a similar manner as cells expressing TSPO1 at the mitochondria^7^,^37^.

Using functional studies we demonstrated that RBCs, which mainly contain TSPO2, are also capable of incorporating the fluorescent haem analogue ZnPPIX. ZnPPIX uptake is enhanced by the addition of TSPO ligands in a concentration-dependent manner and is not related to changes in membrane fragility. It is noteworthy that TSPO ligands promote ZnPPIX accumulation in RBCs, as opposed to cells expressing TSPO1 at the mitochondria, in which TSPO ligands inhibit the intracellular accumulation of haem-related molecules, resulting in a decrease in ROS production.

In agreement with reports of enhanced accumulation of haem-related molecules in RBCs associated with TSPO ligands, we have also demonstrated that ROS production was more rapid in the presence of ZnPPIX. Indeed, the accumulation of ZnPPIX appears to be the link between the effects of TSPO ligands and the increase in ROS generation, leading to the observed inhibition of parasite growth.

ROS-induced stress could result from enhanced ROS production and/or an altered antioxidant cell system. The glutathione system can exert antioxidant effects either by reacting with ROS or by reducing oxidised proteins. Reduced glutathione (GSH) is then transformed into its oxidised form (GSSG) or combined to oxidised proteins (protein-SG). In the present study, the GSSG levels were not significantly changed by the entry of the parasite or the addition of ZnPPIX. Instead, we observed a strong decrease in the cellular content of total glutathione and in the GSH/GSSG ratio in both cases. This decrease is consistent with the use of the antioxidant system to detoxify the ROS that were produced by the uptake of ZnPPIX or by parasite metabolism after the infection of RBCs.

Nevertheless, the entry of ZnPPIX into iRBCs did not demonstrate a synergic effect on the depletion of total glutathione. In the case of iRBCs, the glutathione system manages to buffer the accumulation of ROS induced by the parasite, but the remaining concentration of GSH is too low to buffer the increase in ROS induced by the cellular uptake of ZnPPIX.

To summarise, we have shown for the first time that TSPO ligands promote ZnPPIX uptake in RBCs, acting in an opposite manner to the effects of TSPO ligands observed in mitochondria, where the transport of PPIX is blocked by the addition of TSPO ligands^7^. This difference could be due to the presence of a different TSPO isoform and/or the formation of a different complex in RBC membranes. The PPIX binding site in TSPO2 may differ from the binding site in TSPO1.

TSPO ligands were previously shown to inhibit the ionic conductance activated by parasite infection^17^.

We showed in the current study that TSPO ligands positively modulate the transport of the haem analogue ZnPPIX, leading to increased ROS accumulation. Both effects are not mutually exclusive and could synergistically inhibit parasite growth in RBCs.

Considering that VDAC was previously reported to integrate the new permeability pathway (NPP) assembled after parasite infection^17^ and that TSPO is known to interact with VDAC, we propose that TSPO ligands could modulate the activity of the NPP. However, we cannot discard the possibility that TSPO2 interacts with other RBC proteins and/or parasite proteins exported to the RBC membrane.

Recent studies have provided convincing evidence that the plasmodium CLAG3.2 protein plays a key role in determining the activity of the NPP^40^,^41^. However, this parasite-encoded protein is not homologous to any of the five known anion channel gene families^42^. Although the transmembrane topology of CLAG3.2 is not known, preliminary bioinformatic studies have predicted that the structure of CLAG3.2 exhibits one to three transmembrane domains with a C-terminal transmembrane domain containing negatively charged amino acids, which would be uncommon for anionic channels. These observations lead to the conclusion that if CLAG3.2 participates in the generation of NPPs, it does so as a regulatory subunit rather than as a channel. The assembly of CLAG3.2 monomers into a homomeric or heteromeric complex to form a novel type of anion channel, or the function of CLAG3.2 as an auxiliary subunit in a maxi channel complex involving unidentified parasite-encoded proteins or endogenous RBC channels (such as VDAC) remains to be determined. Further studies are necessary to explore the possible interaction of TSPO2 with proteins in the CLAG3.2 family.

Altogether our results indicate the existence of strong differences between the two TSPO isoforms and the TSPO complexes, leading to different TSPO functions, depending on the location of the isoforms in the RBC membrane or in the outer mitochondrial membrane.

TSPO ligands modulate NNP-driven transport without altering membrane fragility; however, the high ligand concentration and slow kinetics needed to induce ZnPPIX uptake and ROS accumulation does not negate the possibility that ligands could act by targeting proteins other than TSPO2 or VDAC. The design of highly specific TSPO2 ligands is crucial to completely understand TSPO2 function and structure and improve the anti-malarial efficiency of TSPO ligands. Although TSPO1 ligands have been described as neuroprotective agents^43^, TSPO2 ligands may serve as erythroprotective agents and could be useful as an alternative therapeutic strategy to supplement classical anti-malaria drugs.

## Methods

### Chemicals

Otherwise indicated, all chemicals and solvents used were purchased from Sigma–Aldrich (Marne la Coquette, France). Culture media were purchased from Life Technology (Saint-Aubin, France). TSPO ligands (Supplementary Figure 9) were purchased from Sigma-Aldrich (PK 11195 and Ro5-4864) or synthesized following the procedure previously described (SSR-180,575)^44^.

### Antibodies

Polyclonal antibodies used herein were: rabbit anti-hPBR (Santa Cruz Biotechnology, Heidelberg, Germany), rabbit anti-hTSPO2 (Eurogentec, Angers, France) raised against 13 C-terminal residues of hTSPO2 (Supplementary Figure. 3), rabbit anti-hVDAC1, -2, -3 (Santa Cruz Biotechnology), goat anti N-terminus hTSPO1 (Santa Cruz Biotechnology), mouse anti flotillin2 (BD Biosciences, Le Pont de Claix, France), mouse anti-glycophorin A + B (Novus Biologicals, Littleton, USA), FITC-coupled mouse anti-CD61 (BD Biosciences,), APC-coupled mouse anti CD45 (BD Biosciences), PE-coupled mouse anti-glycophorin A (Agilent Technologies, Santa Clara, USA) and rabbit anti spectrin α/β^45^. Immunoflurescence secondary labelling was performed with Alexa Fluor anti –rabbit and –mouse secondary antibodies (Life Technologies). Western Blotting secondary labelling was performed with anti –rabbit, –goat and –mouse secondary antibodies conjugated with horseradish peroxidase (Abliance, Compiègne, France).

### Erythroid differentiation

CD34^+^ progenitor cells were isolated from human cord blood by supermagnetic microbead selection using Mini-MACS columns (Miltenyi Biotec) following the protocol described previously^24^,^25^. Briefly, cells were incubated from days 0 to 7 in erythroid differentiation medium (EDM) supplemented with 1 μM hydrocortisone, 100 ng/mL stem cell factor (SCF), 5 ng/mL IL-3 and 3 U/mL erythropoietin (EPO). From days 7 to 11, GPA positive cells were cultured in EDM supplemented with SCF and EPO. Finally, from days 11 to 15, cells were cultured in EDM supplemented only with EPO. Cells were stained with May-Grünwald-Giemsa reagent for morphological analyses.

### RT-qPCR

mRNA from 10^6^ cells was extracted from cells on the differentiation phase using the RNeasy Mini Kit (Quiagen, Hilden, Germany) as recommended by supplier. 1 μg of RNA was used for cDNA generation using a High-Capacity Reverse Transcription Kit (Life Technologies) as recommended by supplier. Quantitative real-time PCR (RT-qPCR) was performed on 7300 Real Time PCR system (Life Technologies) using a Quantinova SYBR Green PCR Kit (Quiagen) and analysed with the software provided by supplier. RT-qPCR primers were designed using Primer3 software and are listed on Supplementary Table 4. For each primer pair, amplification specificity was validated by melting curve and gel electrophoresis. Relative expression was normalized to unvarying polyadenylate-binding protein 1 (PABPC1) expression.

### Ghost preparation from hRBCs and iRBCs

Samples of concentrated hRBCs that were previously purified using leucodepletion filters, were obtained from the “*Centre National de Référence sur les Groupes Sanguins”* (CNRGS) and washed twice in 1X PBS buffer. Ghosts were obtained by hypotonic lysis performed for 1 h in ice cold 5P8 buffer (5 mM NaH_2_PO_4_; 0.35 mM EDTA, pH 8, containing 1 mM phenyl methane sulfonyl fluoride, PMSF) and centrifuged at 36,000 × g at 4 °C for 30 min as previously described^46^. Several washings with the same buffer were performed until a clear supernatant was obtained. The same procedure was used to obtain ghosts from iRBCs coming from the culture of parasites in basal conditions performed as described above.

The number of platelets and white blood cells (WBC) per million of RBC was measured by FACS analysis using anti-glycophorin A, anti-CD61 and anti-CD45 as specific markers of RBC, platelets and WBC, respectively (Supplementary Table 2).

### Detergent Resistant Membrane (DRM) preparation

DRMs from healthy or infected RBCs were prepared as previously described^47^. Briefly, 2.10^9^ packed erythrocytes were incubated with 1% Triton X-100 in a TBS (25 mM Tris, 150 mM NaCl, 1 mM EDTA) buffer at pH 7.4 containing 1X protease inhibitor cocktail (Roche Innovatis) for 30 min at 4 °C. One volume of 80% sucrose; 200 mM fresh Na_2_CO_3_ in TBS buffer was then added to the RBC extracts and overlaid with 6 ml of a 30% sucrose solution and 2 ml of a 10% sucrose solution in TBS. DRMs were isolated by ultracentrifugation at 225,000 × g for 18 h at 4 °C, in a SW41 rotor. 13 fractions (from the top to the bottom) of 0.9 ml each were collected and further analysed.

### SDS-PAGE and Western Blotting

Ghost samples contained in 5P8 buffer were solubilized in 1% Triton X-100 and ran in an Anykd TGX precast polyacrylamide gel (BioRad). Ghost’s proteins were separated by SDS-PAGE and transferred to nitrocellulose membranes. Immunoblots were performed using the antibodies mentioned above.

### 2-Dimensional Native - SDS PAGE Electrophoresis

Ghost samples contained in 1× 5P8 buffer were solubilized in 1% DoDecylMaltoside (DDM) and ran in a native gradient (3–12%) acrylamide gel electrophoresis (Life technologies) as recommended by supplier. Samples coming from both hRBCs and iRBCs were run in the same 1^st^ Dimension Native – PAGE gel. Lanes of the native gel were excised, treated for alkylation and reduction and ran in a second dimensional 12% acrylamide SDS-PAGE. Western blotting of the samples was performed with the same antibodies described above.

### NanoLC MS/MS analysis

Bands corresponding to immunoblots were excised, and stored in 1% acetic acid before processing.

Samples were treated by Innova Proteomics (Rennes, France). Briefly, samples were successively reduced with DTT (65 mM, 15 min, 57 °C), alkylated with iodoacetamide (135 mM, 50 min, 20 °C), digested with trypsin (10 ng/μl, overnight, 37 °C, Sequencing Grade Modified Trypsin, V511A, Promega). Finally peptides were extracted with Acetonitrile with 0.1% formic acid. Analysis was performed by Innova Proteomics. Mass spectra of VDAC, ANT and TSPO isoforms were obtained using a nanoLC-LTQ-Orbitrap-XL: nanoLC Ultimate 3000 (Dionex) and LTQ-Orbitrap-XL (Thermo Electron).

All spectra were processed by the software Proteome Discoverer 1.0 (Thermo Scientific) with combined analysis via Sequest (Thermo Scientific) and Mascot (Matrix Sciences) and Peaks algorithms for protein identification. Whole identified peptides are included in supplementary dataset 1.

As mentioned above, both the number of peptides detected and the coverage rate are very low compared to those of other well-characterised and abundant proteins.

It is well described that small, highly hydrophobic proteins as TSPO are difficult to detect from classical trypsin digestion in gel without the addition of detergents. Furthermore, in a previous study we have measured the number of VDAC copies to be in the order of 2 × 10^2 ^per RBC^17^.

We used highly purified recombinant human TSPO1 (rec-hTSPO1) to test the canonical mass spectrometry identification protocol. We loaded 100 ng/well of rec-hTSPO1 in a 12% SDS-PAGE gel. After Coomassie Brilliant Blue (CBB) staining, protein bands were excised. In-gel trypsin digestion was performed in the absence or in the presence of 0.01% or 0.1% w/w DDM, followed by nanoLC MS/MS analysis. When DDM was absent the protein was not detectable (0% coverage). The addition of 0.01% DDM permitted the protein detection (53% coverage) raising a maximal coverage (60%) when 0.1% DDM was added (Supplementary Figure 10).

### Immunofluorescence

RBC were washed twice in 1X PBS buffer and fixed in 1% formaldehyde and 0.025% glutaraldehyde for 15 min. After fixing, cells were permeabilized in 1% octylglucoside for 15 min and blocked in PBS containing 1% BSA and 2% Normal Donkey Serum for 20 min. Primary antibodies anti-hTSPO2 (1:200 dilution) or anti-hVDAC1, -2, -3 (1:200 dilution) were added together with an anti-glycophorin A antibody (used as RBC membrane marker) in the same blocking buffer for 1 h. Cells were then washed three times in PBS and incubated for 1 h with the appropriate fluorescently labelled secondary antibody from Life Technologies (1:200). After another set of washes, cells were cytospined onto slides and mounted with ProLong® Gold Antifade Mountant (Life Technologies). Observation was performed using an oil-immersion 63× objective on a laser scanning confocal Zeiss LSM 700 microscope. Negative controls were performed incubating cells in the absence of anti-TSPO2 antibody or saturating the primary antibody with an excess of immunogenic peptide (Supplementary Figure 2).

### *P. falciparum* culture with TSPO ligands

*P. falciparum* strain FCR3 culture was performed in RPMI 1640 medium containing 10% Albumax, at 5% haematocrit, in a 5% O_2_, 3% CO_2_ atmosphere. Cultures were sorbitol-synchronized one parasite life-cycle before experiences were performed. Parasite’s viability assays were started at 1% parasitemia in trophozoite stage; parasitemia was diluted 5-fold (to 1% in control condition) after 48 h for as to allow parasite’s exponential growth throughout the assay. TSPO ligands were added every 48 h (a complete parasite’s life-cycle) throughout the assay together with the renewal of RPMI medium, from ethanol or dimetyl sulfoxide (DMSO) stock solutions at concentrations ranging from 0.1 μM to 50 μM. Control conditions were performed in the presence of the corresponding solvent. Parasitemia was determined after 48 h and 96 h (after one or two complete parasite’s cycles, respectively) by flow cytometry (FACS) (see details below). The haemolysis was evaluated by measuring released haemoglobin as previously described^48^.

### Radioligand binding assays

hRBCs and iRBCs coming from *P. falciparum* cultures (3–5% parasitemia) were washed twice in PBS (pH 7.4). Samples were incubated in a final incubation volume of 0.3 ml, in the presence of (^3^H)PK 11195 (Perkin Elmer, Courtaboeuf, France; 83.5 Ci/mmol) and increasing concentrations of unlabelled TSPO ligands at RT. After 30 min incubation, assays were stopped by filtration through Whatman GF/C filters. Radioactivity trapped on the filters was determined by liquid scintillation counting (Perkin-Elmer, Tri-Carb 2800TR).

### ZnPPIX uptake

*P. falciparum* strains were incubated at 2–5% parasitemia (synchronized cultures, ring and trophozoite stages) in 200 μl of RPMI 10% Albumax containing 20 μM ZnPPIX. TSPO ligands were added at 10 μM and 50 μM. Cultures were kept at 37 °C, in the dark, for 3 to 18 h and further analysed by FACS.

### ROS accumulation

ROS accumulation was measured using the cell permeant ROS probe 2′,7′–dichlorofluorescein diacetate (DCFDA). Cultures pre-incubated with ZnPPIX were washed with PBS and incubated for 30 min in 50 μM DCFDA in PBS (pH 7.2) at 0.5% haematocrit, and kept from O_2_ and light. After washing, DCFDA signalling was assessed by FACS (see details below). Infected RBCs (iRBCs) were differentially stained with the nucleic acid dye TOPRO-3.

### GSH/GSSG ratio determination

Healthy (hRBCs) and iRBCs were incubated for 2.5 h in the presence or absence of ZnPPIX. Total and oxidized glutathione contents were analysed as previously described^4^. Briefly, cells were lysed in a 5% metaphosporic acid buffer and centrifuged at 3,000 × g for 10 min at 4 °C. For total glutathione analysis, supernatant was incubated with glutathione reductase, β-NADPH and 5,5-dithiobis(2-nitrobenzoic acid) (DNTB) for 30 min at 37 °C. For oxidized glutathione analysis, reduced glutathione was blocked by incubation with 10% 2-vinylpiridine for 1 h and samples were further analysed following the same procedure used for total glutathione.

### Fluorescence measurements by flow cytometry

Fluorescence measurements in all samples coming from *P. falciparum*’s viability, ZnPPIX uptake and ROS accumulation assays were performed with a FACSCanto (BD Biosciences) and further analysed with Flowjo-3 software. iRBCs were distinguished from hRBCs contained in the same culture by TOPRO-3 signalling (1/2, 500), with the negative level established with the same non-infected RBCs used for parasite culture. ZnPPIX (Ex.: 488 nm, Em.: 585/42 nm) and DCFDA (Ex.: 488 nm, Em.: 530/30 nm) levels were measured in both infected and healthy RBC populations, results were normalized to the corresponding solvent for each ligand.

### Sorbitol haemolysis assays

For standard semiquantitative haemolysis assays, haemoglobin release was used to estimate lysis time. Culture suspensions (2–5% parasitemia) were washed 3 times in culture medium without serum and resuspended at 50% haematocrit.

Time courses started with the addition of a 0.4 mL aliquot of cell suspension to 3.6 mL of the sorbitol iso-osmotic solutions (300 mM sorbitol, 10 mM Hepes, 5 mM glucose, pH 7.4) to give a cell concentration of 5*10^7 ^cells/mL. Experiments were performed in triplicate.

At predetermined intervals (0, 2.5, 5, 10, 15, 30, 60 minutes), 0.5 mL aliquots of the suspension were transferred to microcentrifuge tubes containing 0.5 mL of ice-cold “stopping solution” (400 mM sucrose in H_2_O). Tubes were centrifuged for 30 seconds. Next, 0.2 μL of the supernatant solution was transferred into 96-well plates for spectrophotometric estimation of haemoglobin concentration by absorption at a wavelength of 540 nm (A540). In all experiments, the A540 value corresponding to full haemolysis of trophozoite-infected erythrocytes was estimated from the final A540 value achieved in the supernatant solution from infected cells suspended in an iso-osmotic sorbitol. When drugs were tested, the percentage of inhibition was determined relative to untreated cells when haemolysis was at maximum. Data analyses were carried out as previously described^49^.

### Ektacytometry Studies

Blood samples were washed three times in PBS and RBCs were resuspended at 3% haematocrit in RPMI medium. RBCs were incubated with 20 μM ZnPPIX with or without 50 μM SSR-180,575 for 8 hours. 80 μL of RBC pellet was used after various incubation time to assess RBC deformability under osmotic gradient by ektacytometry using the Lorrca® Maxsis (Mechatronics, the Netherlands). Studied features from osmotic gradient ektacytometry profiles are O_min_, O_max_, EI_max_ and O_hyper_ as described elsewhere^50^.

### Statistical Analysis

In each graph, unless otherwise noted, data represent the mean ± standard error of the mean (SEM). If indicated, statistical significance has been calculated by one-way analysis of variance followed by Dunnett’s multiple comparison tests. Differences were considered significant when p < 0.05.

## Additional Information

**How to cite this article**: Marginedas-Freixa, I. *et al*. TSPO ligands stimulate ZnPPIX transport and ROS accumulation leading to the inhibition of *P. falciparum* growth in human blood. *Sci. Rep.* **6**, 33516; doi: 10.1038/srep33516 (2016).

## Supplementary Material

Supplementary Information

Supplementary dataset

## Figures and Tables

**Table 1:** Mass spectrometry analysis of TSPO2 and VDAC immunoreactive band, isolated from SDS-PAGE.

Protein	−10logP	Coverage (%)	#Peptides	#Unique	Protein Mass	Best Unique Peptide
TSPO2	34.87	26	1	1	19128.8984	R.DHM(+15.99)SGWCEGPRMLSW(+15.99)CPFYK.V
VDAC1	16.91	15	1	1	30701.5117	K.YQIDPDACFSAKVNNSSLIGLGYTQTLK.P
VDAC2	28.65	27	2	2	31566.5020	K.YQLDPTASISAK(+162.05).V
VDAC3	26.46	22	2	2	30658.6602	K.ASGNLETK.Y
ANT1	30.16	25	5	1	33064.5078	R.I(+162.05)PKEQGFLS(+79.97)FWR.G
ANT2	26.96	42	8	3	32895.2734	K.Q(+162.05)IT(+162.05)ADK(+162.05).Q
ANT3	28.25	41	7	2	32866.2734	K.D(+79.97)FLAGGIAAAISK(+162.05).T
ANT4	26.00	38	4	2	35021.8984	R.EQGFFSFW(+15.99)R.G

**Figure:**