# Supplementary material for: TSPO ligands stimulate ZnPPIX transport and ROS accumulation leading to the inhibition of P. falciparum growth in human blood
Source: Sci Rep. 2016 Sep 19;6:33516. doi: 10.1038/srep33516 (PMC5027585; doi:10.1038/srep33516)
Supplement: Supplementary Information [file srep33516-s1.pdf]

# TSPO ligands stimulate ZnPPIX transport and ROS accumulation leading to the inhibition of *P. falciparum* growth in human blood

Running Title: Mechanisms of P. falciparum inhibition by TSPO ligands

I. Marginedas-Freixa[1], C. Hattab[1], G. Bouyer[4], F. Halle[2], A. Chene[1], SD. Lefevre[1], M. Cambot[1], A. Cueff[4], M. Schmitt[2], B. Gamain[1], JJ. Lacapere[3], S. Egee[4], F. Bihel[2], C. Le Van Kim[1], and MA. Ostuni[1*]

[1]Inserm, UMR-S1134, F-75015 Paris, France; Université Paris Diderot, Sorbonne Paris Cité, UMR_S1134, F-75015 Paris, France; Institut National de la Transfusion Sanguine, F-75015-Paris, France; Laboratoire d'Excellence GR-Ex, F-75015 Paris, France

[2]University of Strasbourg, CNRS, UMR7200, faculty of pharmacy, 67400 Illkirch Graffenstaden, France

[3]CNRS, UMR 7203 LBM, F-75005 Paris, France; Université Pierre et Marie Curie, Sorbonne Universités, CNRS, UMR 7203 LBM, F-75005 Paris, France; École Normale Supérieure - PSL Research University, Département de Chimie

*Corresponding author

**Supplementary Figure 1. Cytological analysis of *in vitro* erythroid differentiation.** CD34+ cells were subjected to *in vitro* erythroid differentiation, following the procedure described by Giarratana et al. (Blood, 2011; Nat Biotechnol 2005), leading to more than 99% erythroid commitment from day 4 of differentiation. Cytological analysis using May Gründwald Giemsa- stained slides were performed at different days after the beginning of differentiation as indicated. Images were obtained from two independent experiments.

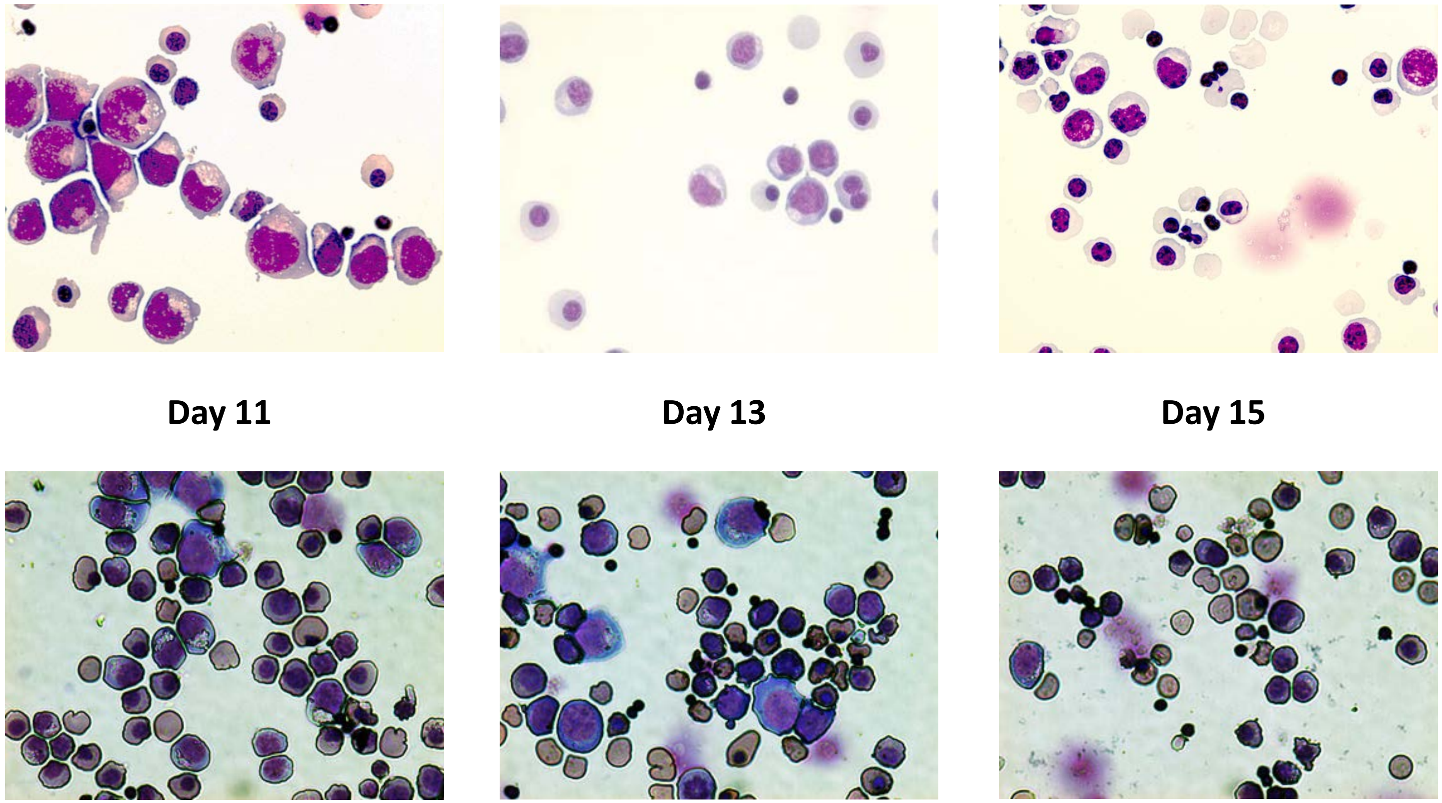

**Supplementary Table 1**. ***In vitro* erythroid differentiation follow-up from $CD34^+$ cord blood cells**. At days 11, 13 and 15 of the cultures, different cell differentiation stages were identified by cytological analysis and quantified. Results of three independent experiments are expressed as the percentage of each cell stage population related to total living cells. Data are presented as mean ± SEM; n=3.

| Day of differentiation | Basophilic | Poly chromatophilic | Acidophilic | Reticulocyte |
|---|---|---|---|---|
| **11 d** | 95.05 ± 4,50 % | 4.95 ± 0.46 % | 0 % | 0 % |
| **13 d** | 80.34 ± 6.59 % | 11.35 ± 0.95 % | 5.28 ± 0,63 % | 3,03 ± 0,55 % |
| **15 d** | 17.55 ± 2.18 % | 28.15 ± 3.11 % | 19.31 ± 2.17 % | 34.99 ± 3.24 % |

**Supplementary Figure 2. Human TSPO isoforms . Sequences alignment localisation of the polyclonal antibody's epitopes**

Human TSPO1 (NM_000714, in black) and Human TSPO2 (NP_001010873, in blue).
Antibodies' epitopes are indicated in underlined bold characters.

1---**mappwvpamg ftl**apslgcf vgsrfvhgeg lrwyaglqkp swhpphwvlg
1------mrlqqgai fvllphlgpi lvwlftrdhm sgwcegprml swcpfykvll

51--pvwgtlysam gygsylvwke lgg-ftekav vplglytgql alnwawppif
48--lvqtaiysvv gyasylvwkd lggglgwpla lplglyavql tiswtvlvlf

100-fgarqmgwal vdlllvsgaa aattvawyqv splaarllyp ylawlafttt
98--ftvhnpglal lhllllyglv vstaliwhpi nklaallllp ylawltvtsa

150-lnycvwrdnh gwrggrrlpe hTSPO1
148-ltyhlwrdsl **cpvhqpqpte ksd** **hTSPO2**

**Supplementary figure 3. TSPO2 is present on RBCs membrane**. Fixed and permeabilized RBCs were incubated in the presence (**A, C**) or in the absence (**B**) of anti-hTSPO2 (1:200 dilution) antibody, in blocking buffer for 1 h. Cells were then washed three times in PBS and incubated for 1 h with the appropriate FITC-labelled secondary antibody from Life Technologies. After another set of washes, cells were cytospined onto slides and mounted with ProLong® Gold Antifade Mountant (Life Technologies). Observation was performed using an oil-immersion 63× objective on a laser scanning confocal Zeiss LSM 700 microscope.  Negative controls were performed without the primary antibody (**B**) or preincubating the primary antibody with an excess of the immunogen peptide (**C**). Bar represent 10 µm

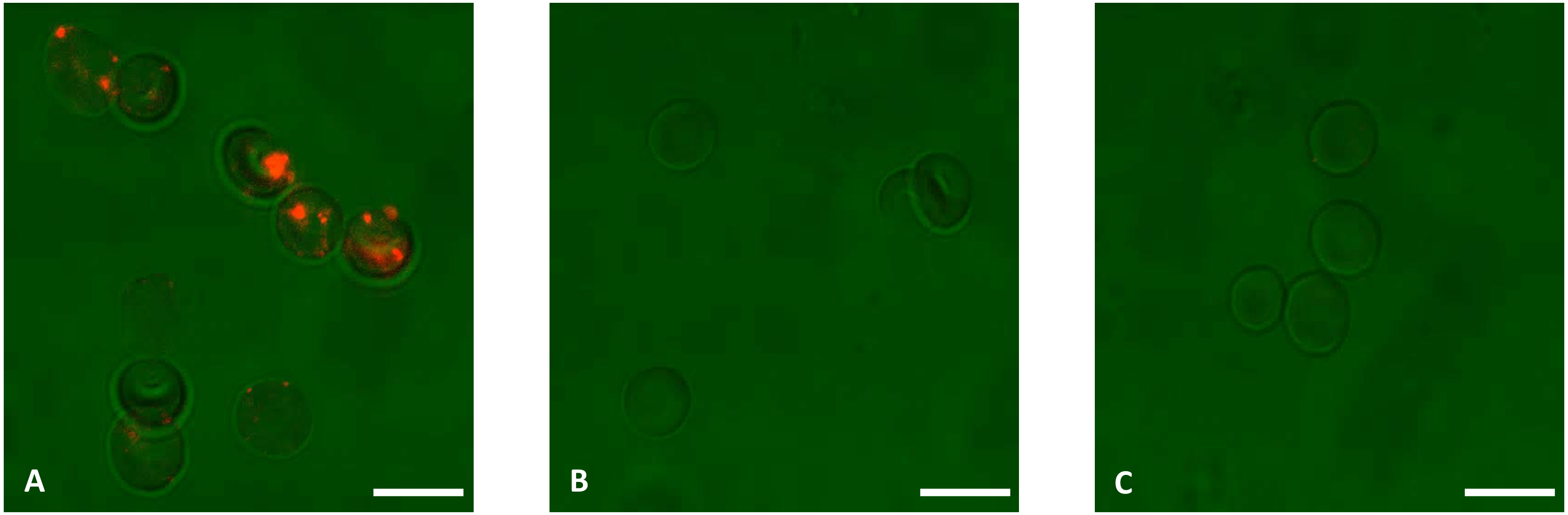

**Supplementary Table 2. Purity of Red Blood Cell Samples.** After leukocyte depletion, concentrated RBC were washed and analysed by FACS using anti-glycophorin A, anti-CD61 and anti-CD45 antibodies as specific markers of RBC, platelets and WBC, respectively. Data are presented as mean ± SEM; n = 6.

| | Before washes | | After 3 washes | |
|---|---|---|---|---|
| **Cell type** | **Events** | **%** | **Events** | **%** |
| RBC | $9.61\ 10^5 \pm 5.43\ 10^3$ | 99.9967 | $9.64\ 10^5 \pm 7.39\ 10^3$ | 99.9975 |
| WBC | 11 ± 1.15 | 0.0011 | 6 ± 2.6 | 0.0007 |
| Platelets | 20 ± 9.9 | 0.0021 | 18 ± 4.9 | 0.0019 |

**Supplementary Figure 4. TSPO2-reactive, high molecular weight band are also recognised by anti-spectin antibody.** Samples of healthy (hRBC) and infected (iRBC) red blood cell membranes (ghosts) were analysed under denaturing conditions after SDS lysis, using anti-spectrin antibody.

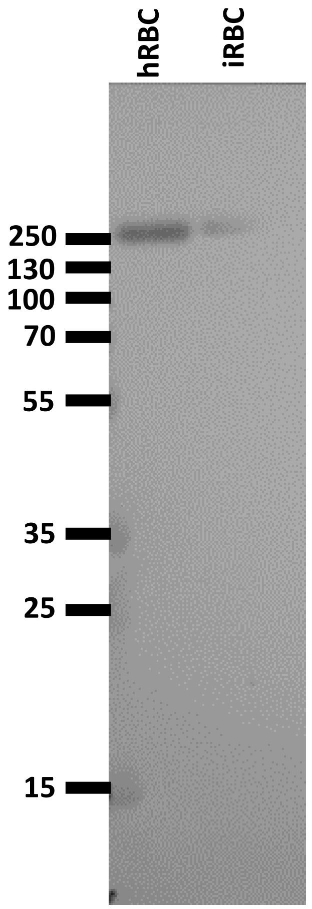

**Supplementary Fig 5. TSPO involving complex on red blood cell membranes.** Samples of red blood cell membranes were solubilized using 1 % DDM and protein complexes were separated under native conditions (BN-PAGE). A 2[nd] dimension denaturing SDS-PAGE was performed and proteins were immunobloted with anti-full length TSPO polyclonal antibody which recognizes both TSPO isoforms. TSPO involving complex migrated at 800 kDa.

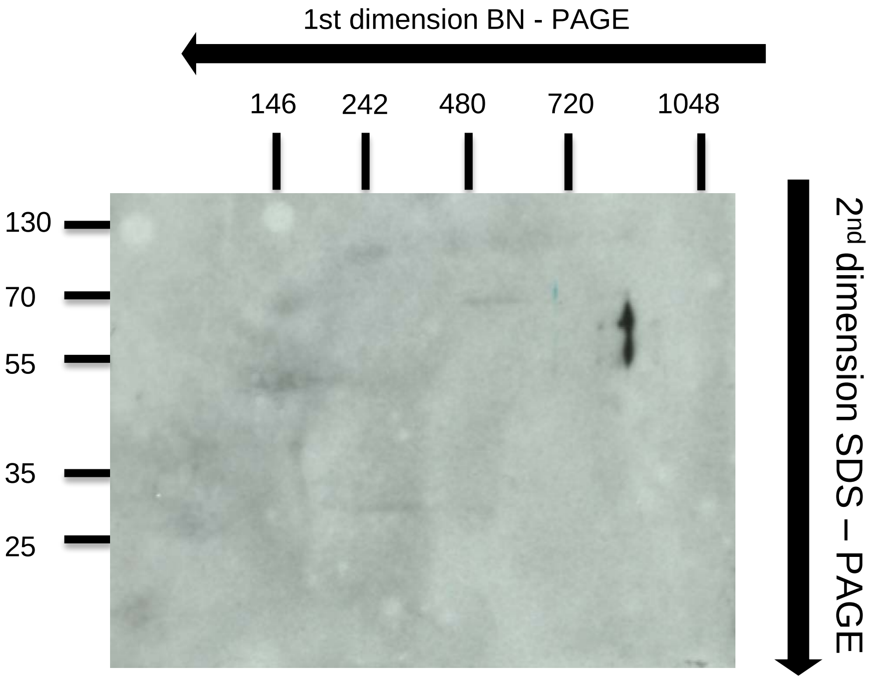

**Supplementary Table 3**. **Effect of ZnPPIX and TSPO ligands on RBCs haemolysis**. RBCs haemolysis was measured in the presence or in the absence of 20 µM ZnPPIX and 50 µM TSPO ligands added to the incubation medium of **(A)** hRBCs or **(B)** iRBC, after 48 hours incubation. Data are expressed as percentage of total haemoglobin released to medium, relative to the total haemoglobin content in samples. Data are presented as mean ± SEM; n = 3.

**(A) hRBCs**

| ZnPPIX | Vehicle | PK 11195 | Ro5-4864 | SSR 180,575 |
|---|---|---|---|---|
| (-) | 0.64 ± 0.02 | 0.60 ± 0.06 | 0.68 ± 0.16 | 0.70 ± 0.08 |
| (+) | 1.13 ± 0.09 | 0.90 ± 0.11 | 1.01 ± 0.07 | 1.01 ± 0.14 |

**(B) iRBCs**

| ZnPPIX | Vehicle | PK 11195 | Ro5-4864 | SSR 180,575 |
|---|---|---|---|---|
| (-) | 0.67 ± 0.04 | 0.81 ± 0.11 | 0.72 ± 0.02 | 0.75 ± 0.11 |
| (+) | 0.83 ± 0.04 | 1.27 ± 0.01 | 1.06 ± 0.08 | 0.96 ± 0.07 |

**Supplementary Figure 6. PPIX uptake is stimulated by TSPO ligands in infected red blood cells (iRBCs).** iRBCs at 2 to 5 % parasitemia were incubated in a media containing 20 µM ZnPPIX and 50 µM TSPO ligands. At several time-points, samples were washed and analyzed in flow cytometry. Data are presented as mean ± SEM; n=4. Differences between each ligand and its control condition (vehicle) were considered significant when *p<0.05; ** p<0.01; ***p<0.001; ****p<0.0001.

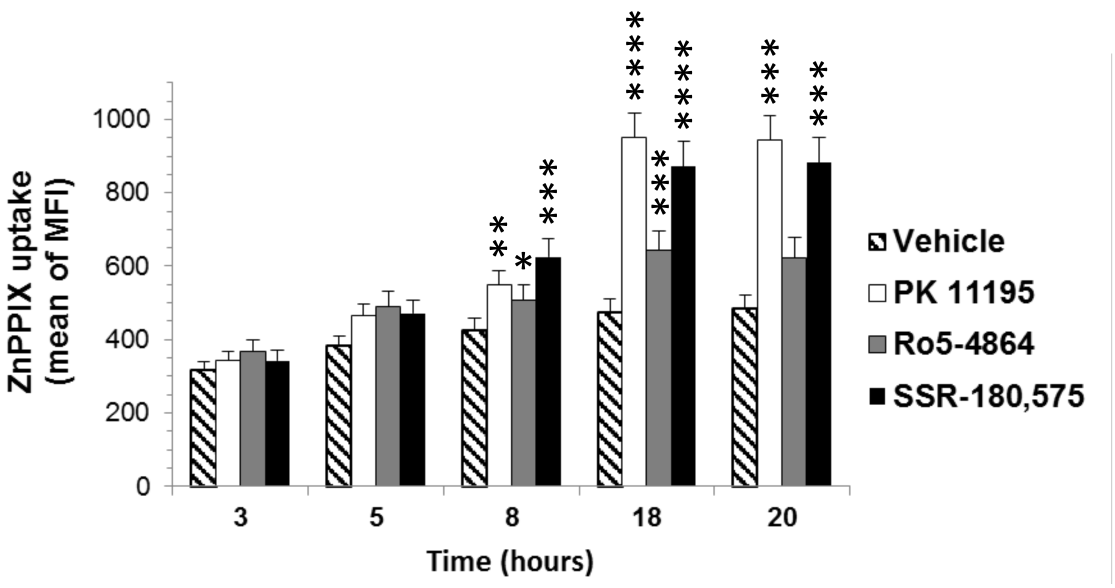

**Supplementary Figure 7. Sorbitol-induced haemolysis is modulated by TSPO ligands in infected red blood cells (iRBCs).** iRBCs at 2 to 5 % parasitemia were washed three times in culture medium without serum and resuspended at 50% haematocrit in a media containing 20 µM ZnPPIX and 10 µM (**A**) or 50 µM (**B**) TSPO ligands. Haemolysis was quantified at several time-points by absorption at 540 nm wavelength. Data are presented as mean ± SEM; n = 4. Differences between each ligand and control condition (vehicle) were considered significant when p<0.01.

A

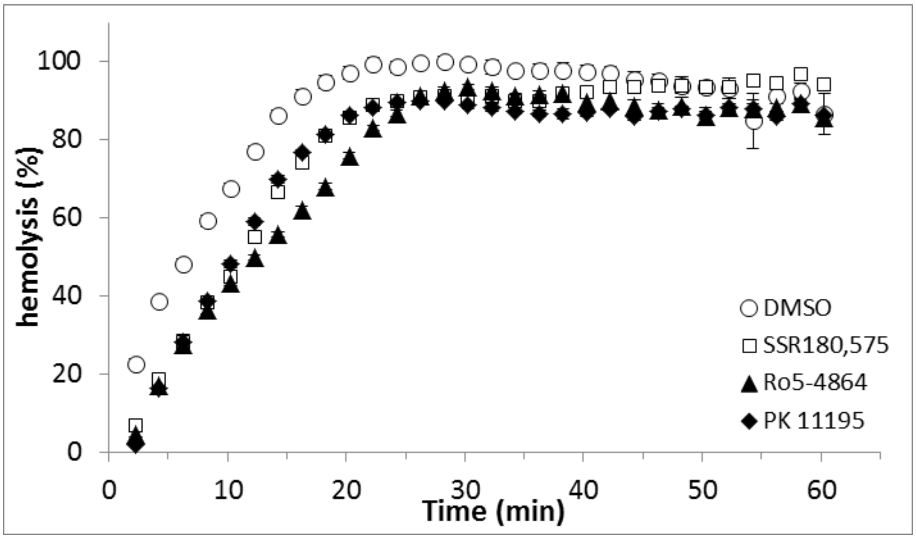


B

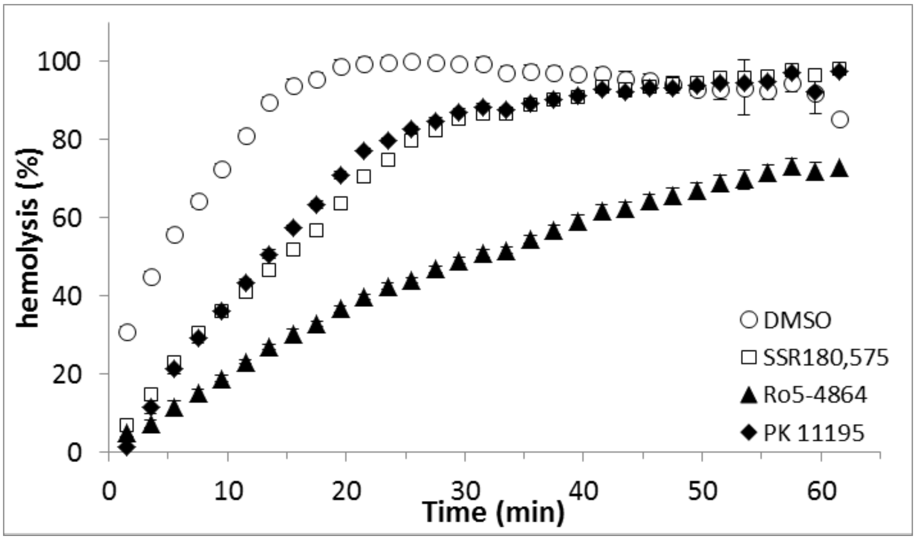

**Supplementary Figure 8. Incubation time to obtain the maximal reactive oxygen species (ROS) accumulation in infected red blood cells (iRBC).** iRBC at 2 to 5 % parasitemia were incubated in a media containing **(A)** 20 µM ZnPPIX and 50 µM TSPO ligands, or **(B)** 50 µM TSPO ligands without ZnPPIX. At several time-points samples were incubated with DCFDA, and analyzed by flow cytometry. The maximum increase on ROS accumulation over the solvent condition is observed at the time point 4.5 hours when ZnPPIX is present in the media and 12 hours when ZnPPIX is absent**.** After the peak, ROS accumulation was strongly diminished. Data are presented as mean ± SEM; n = 4. Differences between each ligand and its control condition (vehicle) were considered significant when *p<0.05; ** p<0.01; *** p<0.001.

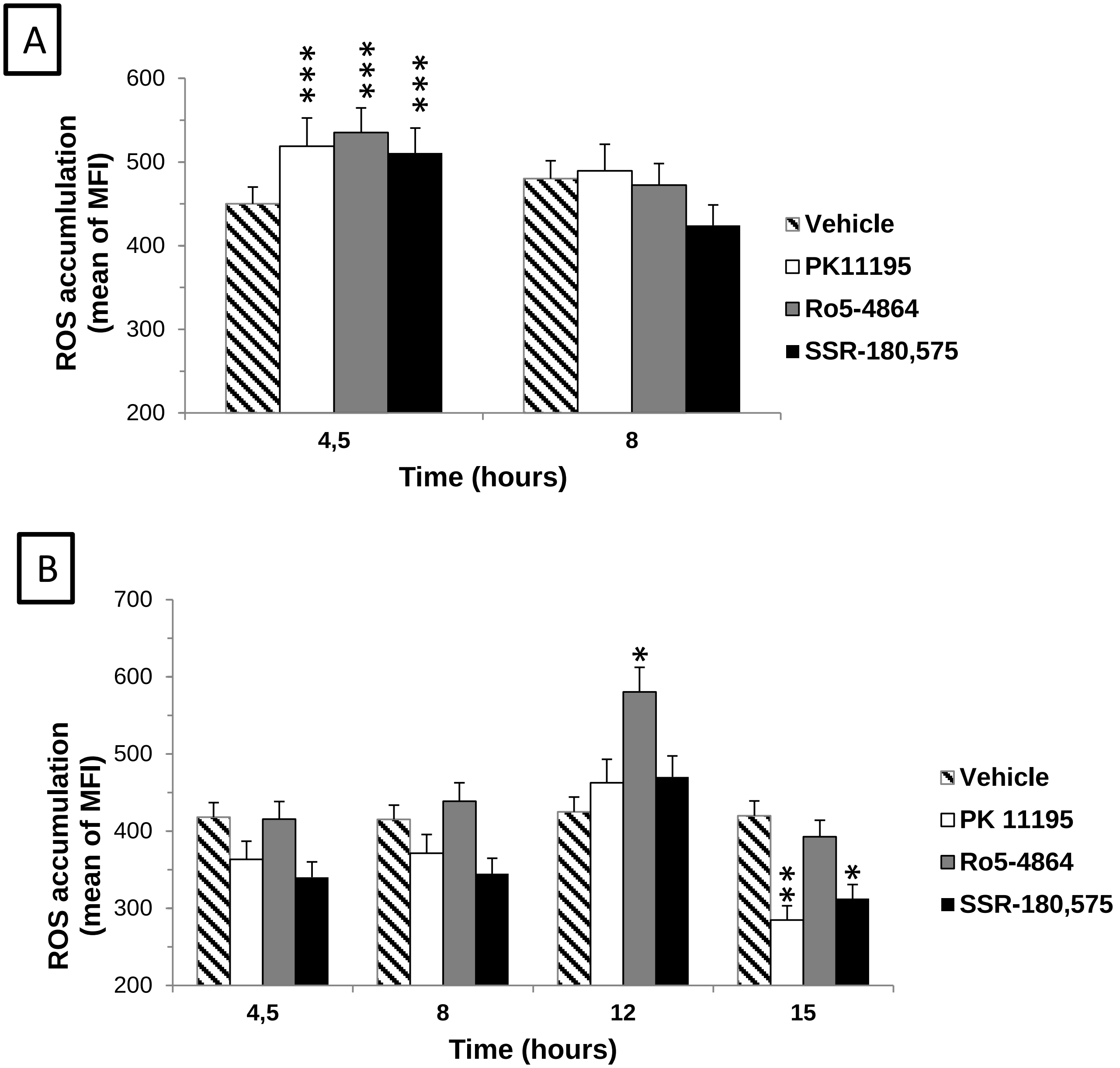

**Supplementary Table 4. Oligonucleotide sequences of the sense and antisense primers used for RT-qPCR experiments**

| Primer | Sequence 5’ – 3’ |
|---|---|
| TSPO1 – S | CTTTGGTGCCCGACAAATGG |
| TSPO1 – AS | CTGACCAGCAGGAGATCCAC |
| TSPO2 – S | TTCACAGTCCACAACCCTGG |
| TSPO2 – AS | AGCCAGTTTGTTGATGGGATG |
| VDAC1 – S | AGCTGACCTTCGATTCATCCTTC |
| VDAC1 – AS | TAATGTGCTCCCGCTTGTACC |
| VDAC2 – S | AGTCTTGCAGTGGCGTGG |
| VDAC2 – AS | TGGTCTCCAAGGTCCCAGTA |
| VDAC3 – S | CAGATGAGTTTTGACACAGCCA |
| VDAC3 – AS | TCCAAATTCAGTGCCATCGTTC |
| PABPC1 – S | GCCACCGGTGTTCCAACT |
| PABPC1 – AS | GCTAGACCTGGCATTTGCTC |

**Supplementary Figure 9. Chemical structure of TSPO ligands**. Chemical structure of the three TSPO ligands used in the present study, belonging to the isoquinolines (PK 11195), benzodiazepines (Ro5-4864) and pyridazinoindoles (SSR-180,575) families.

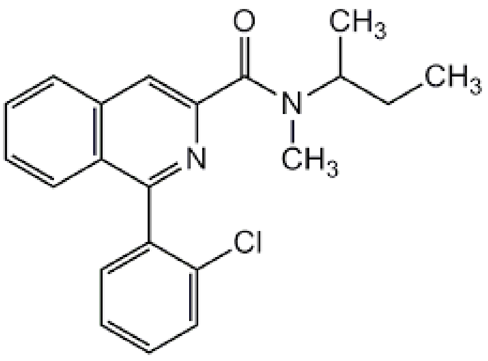


PK 11195

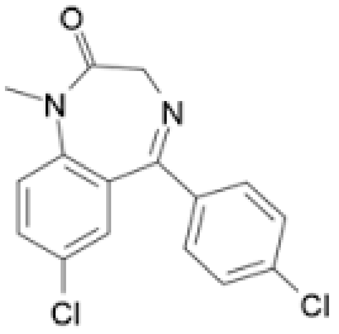


Ro5-4864

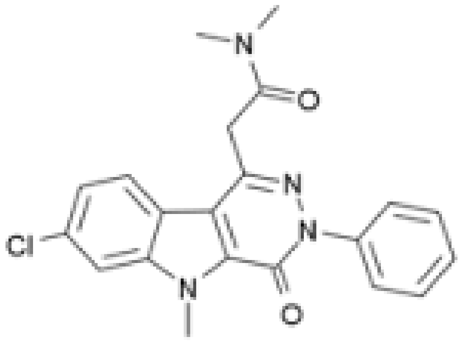


SSR-180,575

**Supplementary Figure 10. Sensitivity of Canonical mass spectrometry identification protocol.** Highly purified recombinant hTSPO1 (hTSPO1rec) was used to test the effect of Dodecyl maltoside on the MS sensitivity to detect TSPO.

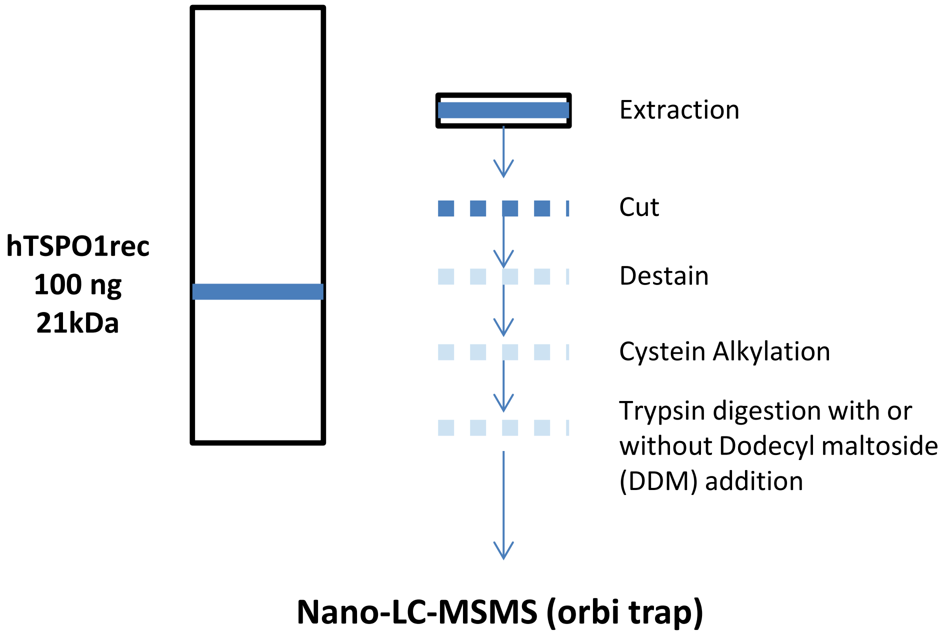


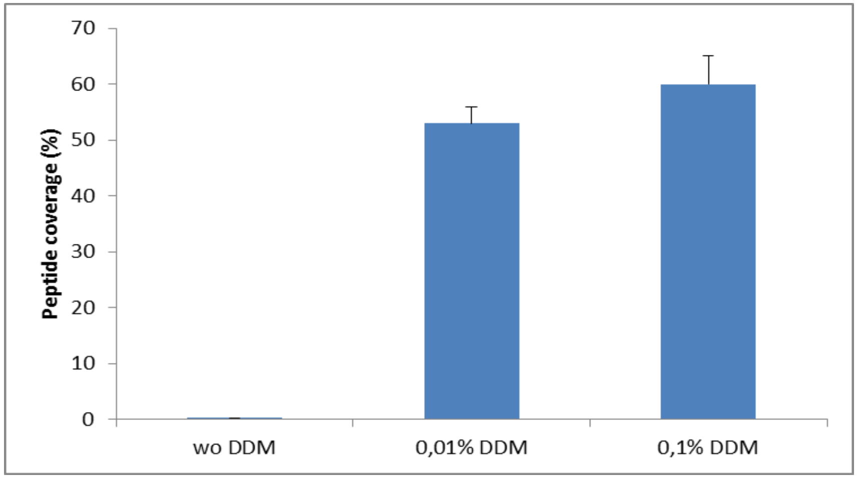


**hTSPO1 sequence (blue bold characters indicate transmembrane domains)**

mapp**wvpamgftlapslgcfvgsrfvhg**eglrwyaglqkpswhpphwv**lgpvwgtlysamgygsylvwke**lgg**ftekavvplg**
**lytgqlalnwaw**ppiffgarq**mgwalvdlllvsgaaaattvawyq**vspl**aarllypylawlaftttlnycvw**rdnhgwrggr
lpe

**Peptides identified after Trypsin + 0,1% DDM digestion**

mapp**wvpamgftlapslgcfvgsr**
**avvplglytgqlalnwaw**ppiffgar
q**mgwalvdlllvsgaaaattvawyq**vspl**aar**
**llypylawlaftttlnycvw**r

**Peptides not identified even after Trypsin + 0,1% DDM digestion**

**fvhg**eglr
wyaglqkpswhpphwv**lgpvwgtlysamgygsylvwk**
**e**lgg**ftek**
dnhgwr
ggrrlpe
